# ‘How to Botox’ on YouTube: Influence and Beauty Procedures in the Era of User-Generated Content

**DOI:** 10.3390/ijerph18084359

**Published:** 2021-04-20

**Authors:** Bárbara Castillo-Abdul, Daniela Jaramillo-Dent, Luis M. Romero-Rodríguez

**Affiliations:** 1 Department of Communication, Rey Juan Carlos University, 28942 Madrid, Spain; prof.bcastillo@eserp.com; 2 ESAI Business School, University Espiritu Santo, Samborondon 092301, Ecuador; 3 Department of Education, University of Huelva, 21007 Huelva, Spain; daniela.jaramillo@pi.uhu.es; 4 Faculty of Communication & Arts, Nebrija University, 28015 Madrid, Spain

**Keywords:** YouTube, social networks, influencers, Botox, tutorial, social media

## Abstract

The current media environment is complex and has important effects on all aspects of life, including beauty and health. In this sense, YouTube has become one of the main contexts for the dissemination of tutorials and content related to medical procedures such as the application of Botox. Thus, the present study constitutes the first exploratory analysis of YouTube videos in Spanish related to this procedure. A preliminary analysis of 221 YouTube videos yielded a final sample of the 50 most viewed videos within this genre. The analysis was carried out through a quantitative content analysis assessing the popularity of the videos, contact and emotive strategies by the creator, the credibility conveyed, and the characteristics of information about the procedure itself. Results suggest that these influencers align with mainstream Internet celebrity culture in practices that aim at increasing their following and views, as well as calls for subscriptions and visits to other platforms and profiles. Moreover, they include different strategies to establish their credibility but emphasize personal experience. The positive portrayal of the procedure, including positive emotions and content that highlights the benefits, is interesting and supports the commercial nature of much of the content.

## 1. Introduction and State of the Art

Current media configurations empower consumers to turn into producers of content, in many cases, with the aim of harnessing a relevant following [1], to like, comment, and share their productions in a process enabled by social networking sites and their unique functionalities. In this environment, YouTube rises as one of the leading platforms for participation [2], with more than two billion users [3] in a digital context where ordinary people can become micro-celebrities or so-called influencers [1,4,5].

In this ecosystem, information sources and genres converge, making it increasingly difficult for users to identify and discern reliable messages from inauthentic ones [6]. This includes the ability to differentiate scientific information from opinions and advertising [7]. The described phenomena are of great concern in general, but they are especially troublesome when affecting information related to health and medical procedures.

Considering that social media is one of the primary sources for cosmetic surgery-related content including non-invasive procedures like Botox injections [8], it is relevant to assess the characteristics of such content. Thus, the present study aims to evaluate the characteristics of YouTube videos in Spanish that feature Botox-related content.

Furthermore, YouTube has become the second most popular social media platform worldwide [9]. It has risen as the leading social media site for user-generated content creation, modification, and dissemination, with beauty vlogging taking center stage [10,11]. This develops in a context that places an ever-increasing emphasis on physical appearance, defined by representations of beauty perpetuated through both conventional and social media. Thus, the emergence and success of beauty influencers on YouTube have solidified the focus of the current media ecosystem on ideal physical traits, serving as the perfect platforms for product marketing [12]. The beauty influencer market is massive; in 2019, it was calculated at $8 billion worldwide [13], suggesting that it is an area with great monetization possibilities and one that is highly valued by companies attempting to persuade users to purchase and use their products, including products like Botox [14].

Moreover, the functionalities and practices of users and influencers in the digital realm have adapted to the need to look perfect, with an increasing focus on the use of filters, image enhancement, and digital manipulation to appear closer to these ideals. Society has never appeared more beautiful, more standardized, or more filtered. Meanwhile, the same digital environment that has helped define what is acceptable in terms of aesthetic traits has become a prime context for the creation and dissemination of a variety of content relating to beauty [10]. YouTube is the perfect platform due to the ability of creators to upload content to an account, gain followers, and interact with them through comments, shares, likes/dislikes, and views. The ideal of beauty in the era of social media involves skin that is free from lines, scars, and the natural aging process, a skin attainable through the use of a variety of products; among these, one of the most popular is Botox [14].

Studies attempting to assess YouTube videos as health information sources have focused on diverse types of content, signaling some of the most problematic issues in the health information sphere today. These include the exploration of claims made in pro- and anti-vaccination content on the video-sharing platform [15]; other studies have focused on pro-anorexia, also known as pro-ana productions, with findings that suggest that the YouTube algorithm yields this content even in cases when the search is aimed at finding help for eating disorders [16]. Moreover, the high popularity of anti-vaccination and pro-ana videos has been evidenced in both cases, as this problematic content displays higher ratings than other types of anorexia- and vaccination-related content [16,17]. More recently, studies have focused on COVID-19 content and confirmed the presence of misleading information among the most viewed videos [18], while findings suggest that independent users are more likely to post misleading videos, while news organizations are more likely to upload useful videos [19]. In the case of YouTube videos in Spanish, information is often incomplete, and the type of authorship has an effect on the reliability of the information offered [20].

Research aimed at examining beauty procedures on YouTube reflects the range of audiovisual productions available and their characteristics. An exploration of lip filler mentions within videos on the social media site is present in content by eight of the 10 most popular beauty influencers [21]. Moreover, in an analysis of cosmetic surgery videos, researchers found that most of them emphasized the positive aspects of procedures rather than their risks, both in expert content and user-generated productions [8]. In terms of the evaluation of YouTube videos related to genioplasty, these have been found to be inappropriate as a source of information for patients [22].

Botulinum toxin type-A (known by its commercial name Botox) injections are some of the most popular cosmetic procedures worldwide for the treatment of wrinkles [14]. Videos featuring this procedure are widely available on YouTube and have been found to be useful for users looking to make a decision on whether to undergo this intervention [6]. Moreover, celebrity recommendations and Botox tutorials modeling how to apply the product reflect its popularity among YouTube users [14]. Along the same lines, DIY Botox application practices have also been documented and explored in online forums, where users recommended YouTube tutorials and injection maps to self-apply the product [23]. Researchers have also analyzed the quality of YouTube videos featuring Botox application for patients with spasticity, with results that suggest that the videos that appear on the first page of the YouTube search are not the most reliable and useful but the ones with the most views [24]. Finally, the typologies of Botox videos have been examined, suggesting that the most viewed content included patient experiences, advocacy videos, and live procedures [25].

As most previous research has focused on English content, it becomes necessary to explore content in Spanish. This study fills the existing gap in the literature by focusing on Spanish content. The present analysis assesses three aspects: the relationship established between the YouTuber and the viewer, the strategies used to convey credibility, and the framing of the specific procedure. Thus, four research questions guide this study: (RQ1) What are the strategies used most often by Spanish-speaking YouTubers to promote a relationship with their viewers? (RQ2) What approaches do YouTubers deploy in their videos to establish their credibility within Botox-related content in Spanish? (RQ3) What are the characteristics of Botox procedures emphasized by Spanish-speaking YouTubers? For the purposes of the following question and sub-questions, interactions are defined as follows, likes, dislikes, views, and comments. The duration of videos is also included as a significant variable within the general characteristics of the video. (RQ4a) What are the differences in terms of interactions and video duration among YouTube videos that employ strategies to promote a relationship with their viewers? (RQ4b) Credibility strategies? (RQ4c) Positive and negative perspectives about Botox?

## 2. Materials and Methods

The present study has an exploratory–descriptive scope, insofar as the phenomenon of apomediated prescribers of cosmetic treatments through the application of Botox in YouTube videos in Spanish has not been sufficiently addressed by the scientific community. In this sense, the present exploratory research seeks to gain a deeper understanding of scarcely known phenomena with the intention of establishing variables, dimensions, and indicators of interest for future studies [26].

For this purpose, an interpretive quali-quantitative content analysis was performed on 50 YouTube videos published in Spanish, with the following sampling criteria: (1) the videos had to be uploaded in the last five years (2017–2021); (2) they had to be uploaded by non-professional channels (not specialized in official medical or cosmetic information); (3) the videos had to include a demonstration related to the application of Botox treatment injections on oneself or others. Using these criteria, a total of 221 videos emerged, which were organized by number of reproductions. The final sample consists of the 50 videos with the highest number of views.

An analysis sheet, a theoretically derived codebook duly validated in construct and content by expert judgment, was used to collect the information, consisting of seven dimensions, 25 categories, and 75 indicators (see Table 1). The dimensions were derived from previous literature, and they include contact enunciation, emotive enunciation, and genre [27]. Additionally, three categories specific to medical information and Botox were incorporated: strategies of credibility and reliability, attitudes towards Botox, and specific content about Botox [6]. The average level of inter-rater agreement ($\bar{X}$) was 4.2 (on a Likert scale from 1—strongly disagree to 5—strongly agree) and Cohen’s Kappa (*k*) was 0.770.

For the analysis of the 50 videos mentioned above, which make up the study sample of this exploratory research, three coders—the same authors of the study—defined each of the dimensions and indicators before coding to standardize the interpretations of the qualitative analysis. It is important to note that none of the videos studied contained legal disclaimers.

We studied the frequency distribution and the descriptive values of all the variables of the study, including skewness and kurtosis for the general variables, to observe whether they were normally distributed. Then, we conducted correlations (Pearson’s R) between the general variables and between these and the dichotomous variables. All of them, when significant, were accompanied by dispersion diagrams to visually illustrate the results.

Finally, to observe how the dichotomous variables relate, we conducted crosstabs and calculated Chi Square (x^2^) to test whether they significantly relate to each other. Given that they are dichotomous variables transformed into dummy ones for their analysis, this could have been done with correlations and results would have been similarly interpreted, but Chi Square (x^2^) is considered more adequate. Given the lack of balance in most variables, these tests did not offer many relevant results, although some exploratory observations were considered interesting and might be deepened in future studies.

## 3. Results

### 3.1. Frequencies and Descriptive Values of General Variables

There is concentration in smaller values, that is, the curves are strongly right-skewed. Videos are rather popular: the mean number of followers is 92,459.9 (SD = 138,769.406), the mean number of likes is 13,389.12 (SD = 29,069.475), the mean number of dislikes is 642.68 (SD = 1697.49), the mean number of comments is 956.2 (SD = 2056.505), and the mean number of views is 412,584.64 (SD = 830,935.137). Those are, in general, high values (Table 2), but the mean should be carefully interpreted in this case, because the standard deviation, skewness, and kurtosis reflect very high values, showing how most videos are located in small figures, but some very influential videos might be strongly affecting the mean. However, video duration shows a slightly different path, with a mean of 15:03 min (SD = 06:05 min) and a much lower skewness and kurtosis, although still concentrated on the lower values.

### 3.2. Frequencies of Dummy Variables

Many of these variables are present in all the videos, which makes it impossible to conduct any further analysis for them. For example, the target audience is always the general population and never professionals, which is expected, considering the sampling criteria included only videos uploaded by general YouTubers rather than professionals and official accounts. Additionally, in response to RQ1, the speaker is never absent from the camera while the voice can be heard, while in only 6% of the cases there is on-camera presence and voice but not addressing the camera directly, and in all the other cases the person looks and speaks directly into the camera. Except for 8% of the videos, in most of them, the YouTuber uses the second person and its variations to relate to the public, while in 62% of the videos the speaker(s) directly requests comments and/or opinions, and in only one case (2%) the name of a follower is used, and there is also one case where there is a response to a specific follower’s comment. Additionally, in 82% of the cases, they use phrases to promote subscriptions, 94% of the videos feature invitations to watch other videos on the channel, and 98% include invitations to visit accounts on other platforms.

**Figure 1:**
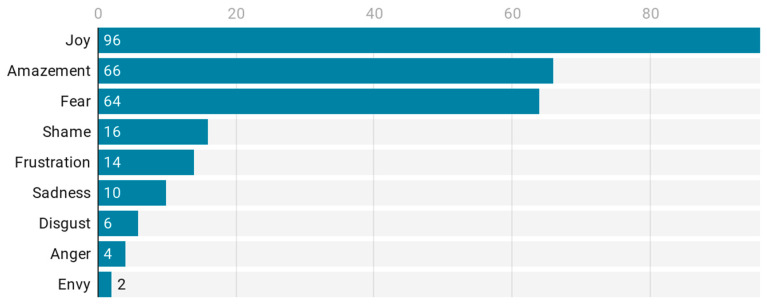
Emotional expressions.

The emotional expressions tend to be positive: thus, joy is present in 96% of the videos and amazement in 66%. Meanwhile, sadness is present only in 10%, anger in 4%, shame in 16%, frustration in 14%, disgust in 6%, and envy in 2%. The only exception in terms of negative emotions is fear, which is present in 64% of the videos. This is clearly connected to the emotional appeal towards the follower/user, which appears in 96% of the videos. Emotional appeals include joy, which is used in 88% of the cases, and amazement in 66%, while sadness is only used in 6%, anger is never used, shame is used in 2%, frustration in 16%, fear in 14%, disgust in 4%, and envy in 4% (see Figure 1).

The tutorial explains the complete process in 94% of the cases, and there is information about the products necessary to carry out the procedure in 98% of the cases. Videos include a brand in 40% of the cases, and 82% of them mention a specific center or professional, while 66% of the videos include a promotional message to sell a product.

**Figure 2:**
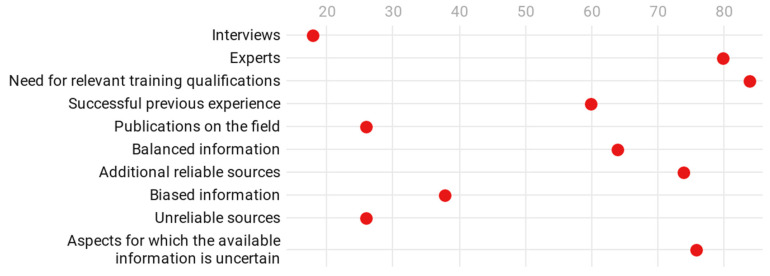
Information quality about the treatments.

Also in relation to RQ1, and in terms of the mention of information without appealing to the user to take any action, this only happens in 16% of the videos, while 18% of them feature an interview with a person external to the YouTube channel, and 80% include an expert on the subject. All the videos develop first-person content. In response to RQ2, and in terms of personal expertise, 84% of the videos mention relevant training or qualifications, 60% mention successful previous experience, 26% mention publications on the subject, and all of them explain that the information is based on the personal experience of the YouTuber. Furthermore, 64% of the videos show balanced information, 36% show biased information, and 74% include additional reliable sources, while 26% include sources that are unreliable or impossible to know. Furthermore, 76% of the videos mention aspects for which the available information is uncertain (see Figure 2).

In terms of the attitude towards Botox, and in response to RQ3, 92% of the videos describe positive aspects of the product, 42% mention negative ones, and 42% include both positive and negative aspects of the product. In 90% of the cases, there is an explanation about how the product works, while none of them include a description about how to apply the product that is directed at professional target audiences, and in one case (2%) there is a description of how the product is applied (for self-application). In 34% of the videos, application “maps” are used. All the videos include a description of the benefits that can be expected, while only 44% mention side effects, 26% describe contraindications, and 34% feature a description of possible risks.

Finally, regarding self-care after application, half of the videos include instructions, 20% mention things that can be done, and 32% describe things that should not be done.

### 3.3. Correlations between General Variables

**Figure 3:**
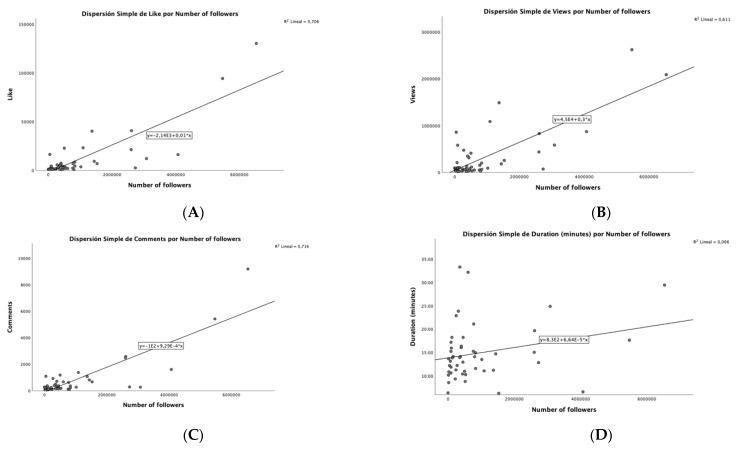
Dispersion diagrams of correlation between n followers and interactions. Note: From up to bottom and left to right: (**A**) dispersion between n likes and n followers; (**B**) dispersion between n views and n followers; (**C**) dispersion between n comments and n followers; (**D**) dispersion between duration (in minutes) and n followers.

There is a strong positive correlation between all variables (for example, the more followers, the more likes, dislikes, views, and comments), except for the duration, which has a significant positive correlation with the number of likes, dislikes, and comments, although the size is smaller. All these correlations can be seen more clearly in the dispersion diagrams (Figure 3).

## 4. Correlation between General Variables and the Non-Constant Dummy Variables

In this section, the results respond to RQ4 and related sub-questions. In terms of RQ4a, the number of followers is significantly correlated with the use of the name of a follower (R(49) = 0.589, *p* < 0.001), the expression of anger (R(49) = 0.372, *p* < 0.01), the expression of envy (R(49) = 0.478, *p* < 0.01), the emotional appeal to sadness (R(49) = 0.339, *p* < 0.05), the emotional appeal to shame (R(49) = 0.589, *p* < 0.001), the emotional appeal to envy (R(49) = 0.467, *p* < 0.01), and the inclusion of promotional messages to sell a product (R(49) = −0.319, *p* < 0.05). Except in the last case, the other are present in very few cases, so these correlations must be carefully interpreted.

The number of likes significantly correlates with the on-camera presence and voice but not with addressing the camera (R(50) = 0.291, *p* < 0.05), the use of the second person (R(50) = −0.358, *p* < 0.05), the use of the name of a follower (R(50) = 0.579, *p* < 0.001), the expression of anger (R(50) = 0.386, *p* < 0.01), the expression of envy (R(50) = 0.401, *p* < 0.01), the emotional appeal to sadness (R(50) = 0.306, *p* < 0.05), the emotional appeal to shame (R(50) = 0.579, *p* < 0.001), the emotional appeal to fear (R(50) = 0.412, *p* < 0.01), or the emotional appeal to envy (R(50) = 0.314, *p* < 0.05). In response to RQ4b, the number of likes significantly correlates with the mention of relevant training or qualifications (R(50) = −0.355, *p* < 0.05), as to RQ4c, the number of likes correlates with the description of negative aspects of the product (R(50) = 0.304, *p* < 0.05) and the inclusion of positive and negative aspects of the product (R(50) = 0.343, *p* < 0.05). In this sense, it is noteworthy that mentions of relevant training or qualifications present a significant negative correlation with the number of likes, in response to RQ4b.

Furthermore, and related to RQ4a, the number of dislikes significantly correlates with on-camera presence and voice but not with addressing the camera (R(50) = 0.317, *p* < 0.05), the use of the second person (R(50) = −0.370, *p* < 0.01), the use of the name of a follower (R(50) = 0.726, *p* < 0.001), the expression of anger (R(50) = 0.505, *p* < 0.001), the emotional appeal to sadness (R(50) = 0.402, *p* < 0.01), the emotional appeal to shame (R(50) = 0.726, *p* < 0.001), or the emotional appeal to fear (R(50) = 0.484, *p* < 0.001); in relation to RQ4c, the number of dislikes significantly correlates with the description of negative aspects of the products (R(50) = 0.283, *p* < 0.05) and the inclusion of positive and negative aspects of the product (R(50) = 0.288, *p* < 0.05).

In response to RQ4a, the number of views has a significant correlation with the on-camera presence and voice but not with addressing the camera (R(50) = 0.386, *p* < 0.01), the use of the second person R(50) = −0.503, *p* < 0.001), the use of the name of a follower (R(50) = 0.291, *p* < 0.05), the emotional expression of envy (R(50) = 0.383, *p* < 0.01), the emotional appeal to shame (R(50) = 0.291, *p* < 0.05), or the emotional appeal to fear (R(50) = 0.401, *p* < 0.01). Furthermore, in relation to RQ4b, the number of views significantly correlates with the inclusion of an expert on the subject (R(50) = −0.280, *p* < 0.05), the mention of relevant training or qualifications (R(50) = −0.414, *p* < 0.01), the use of balanced information (R(50) = −0.311, *p* < 0.05), the use of biased information (R(50) = 0.291, *p* < 0.05), the description of negative aspects of the product (R(50) = 0.334, *p* < 0.05), and the inclusion of positive and negative aspects of the product (R(50) = 0.370, *p* < 0.01). It should be highlighted, like in the case of likes, that the inclusion of an expert, the mention of relevant training or qualifications, and the use of balanced information have a negative correlation with the number of views; however, it should be noted that correlation does not mean causality, and this negative correlation should be further explored.

In terms of RQ4a, the number of comments significantly correlates with on-camera presence and voice but not with addressing the camera (R (50) = 0.340, *p* < 0.05), the use of the second person (R (50) = −0.345, *p* < 0.05), the use of the name of a follower (R(50) = 0.577, *p* < 0.001), the emotional expression of anger (R (50) = 0.410, *p* < 0.01), the emotional expression of envy (R(50) = 0.312, *p* < 0.05), the emotional appeal to sadness (R(50) = 0.333, *p* < 0.05), the emotional appeal to shame (R (50) = 0.577, *p* < 0.05), the emotional appeal to fear (R (50) = 0.458, *p* < 0.05), or the emotional appeal to envy (R(50) = 0.299, *p* < 0.05). Moreover, in response to RQ4b, the number of comments significantly correlates with the mention of relevant training or qualifications (R (50) = −0.331, *p* < 0.05), and in relation to RQ4c, the number of comments significantly correlates with the description of negative aspects of the product (R (50) = 0.292, *p* < 0.05) and with the inclusion of positive and negative aspects of the product (R (50) = 0.311, *p* < 0.05).

In relation to RQ4a, the duration of the video significantly correlates with the use of the name of a follower (R (50) = 0.340, *p* < 0.05), the emotional expression of fear (R(50) = 0.288, *p* < 0.05), the emotional appeal to shame (R (50) = 0.340, *p* < 0.05), and the emotional appeal to amazement (R (50) = −0.303, *p* < 0.05), and in response to RQ4b, video duration correlates with the inclusion of a person external to the channel in interview format (R(50) = 0.361, *p* < 0.05) and with the explanation of how the product works (R (50) = 0.307, *p* < 0.05).

Although the duration seems to follow a slightly different path, in general, many significant correlations are present with the same few variables, mostly in cases where just very a few of the videos differ from the majority, which seems to have a strong effect (if only one or two videos include one aspect and that happens in videos with a lot of views, there might be a strong correlation even if it might not be related). This can be seen in the diagrams, in which the correlation line is only based on one of very few values. Similarly, given the strong correlations between the general variables, it does make sense that they also show similar patterns with the dichotomous variables (if the videos in which one aspect is present are highly viewed, and therefore there is a correlation between the number of views and that aspect, it is likely that they will also have a large number of likes, and there will be also a correlation between the number of likes and that aspect).

Although partially explained by these remarks, it should be noted that, maybe surprisingly, and in response to RQ4a, the presence of negative emotional expressions and appeals seems to lead to higher figures in terms of views, likes, or followers, while the opposite happens in relation to RQ4b, with the mention of training or qualifications or of balanced information. Although this observation does not imply causality, its interest might make it relevant to further analyze it.

## 5. Exploratory Relations between Dichotomous Variables

We have conducted some exploratory Chi-square tests between the dichotomous variables to observe whether they are related (Chi-square is more useful than correlation for this, but the interpretation and the results are very similar). However, many of these variables were constant or had very few cases in one of the options and a lot in the other ones (depending on the case, the absence or the presence is strongly dominant and only one or two cases are different), and this makes it harder to find clear patterns. For this reason, there are almost no relevant results here: the emotional expression of disgust is significantly more probable when there is no emotional expression of joy, for example, but only three videos showed disgust and only two did not show joy, so the results here are not very strong. Something similar happens with the rest of the expressions of emotions and emotional appeals, with only two exceptions that could be mentioned: The expression of amazement, in which there is also a more balanced distribution, values were not significant, except regarding the expression of frustration, as both emotions were significantly more present when the other also appeared [2.0 > 1.96; X^2^ = 4.193, *p* < 0.05); the expression of fear, as once again, both feelings were significantly more present when the other also appeared [3.0 > 1.96; X^2^ = 9.212, *p* < 0.01); and the emotional appeal to amazement, which again was significantly more present when both forms of amazement appeared together [3.3 > 1.96; X^2^ = 10.822, *p* < 0.01). The expression of fear, which also had a more balanced distribution, was significantly more present when there was also an expression of frustration [2.1 > 1.96; X^2^ = 4.578, *p* < 0.05), and also when there was an emotional appeal to fear [2.1 > 1.96; X^2^ = 4.578, *p* < 0.05).

Another pair that offers more balanced distributions between absence and presence is the relationship between the direct request of comments and/or opinions (19 absence/31 presence) and the use of phrases to promote subscriptions (9/41): it is significantly more likely to include one when the other is also present; however, the *p*-value is exactly 0.05, so these are not strong results.

A very clear case is the interconnection between the description of contraindications and the description of possible risks, which are positively related [5.2 > 1.96; X^2^ = 26.616, *p* < 0.001), and both the description of side effects [4.5 > 1.96; X^2^ = 20.455, *p* < 0.001) and the description of contraindications [3.4 > 1.96; X^2^ = 11.761, *p* < 0.001) are significantly more present together with the description of side effects. These are the three most clearly significant relations of the study.

The relationship between mentioning having had experience with successful cases and mentioning publications on the subject is also significantly positive [2.1 > 1.96; X^2^ = 4.435, *p* < 0.05). Finally, the presence of a brand is significantly less common when an expert is present in the video [2.9 > 1.96; X^2^ = 8.333, *p* < 0.01) when relevant training or qualifications are mentioned [2.2 > 1.96; X^2^ = 4.861, *p* < 0.05) and when both positive and negatives aspects of a product are mentioned [2.0 > 1.96; X^2^ = 3.955, *p* < 0.05).

## 6. Discussion

The purpose of this study was to explore the characteristics of apomediated YouTube videos in Spanish about Botox application to assess their characteristics. In the next sections, a discussion of the results will be presented.

### 6.1. Contact and Emotive Enunciation

The results of the present study, namely the fact that 100% of the videos are directed towards the general population rather than professionals, suggest that YouTube constitutes a space where medical information, including highly specialized procedures such as the application of Botox, is discussed directly with users, often bypassing expert advice and fulfilling the motivations of users to consume health information on YouTube [28]. Although the sampling criteria excluded videos created by professionals, it is relevant to mention that user-generated content related to Botox application is aimed at the general population rather than professionals, because it supports previous research as explained above and sets the stage for the analysis of the relationship-establishing strategies that respond to RQ1. In response to RQ1, YouTubers reflect the use of linguistic strategies that constantly establish contact between the viewer and the YouTuber. In this sense, and across the sample, contact enunciation codes involve practices such as the use of the second person in 92% of the videos and speech directed at the camera in the vast majority of videos, illustrating the constant contact with followers and viewers in general [27].

Other strategies deployed in these videos that respond to RQ1 include the use of verbal strategies to promote further actions from viewers such as follows, views, and subscriptions to other channels and profiles in other platforms, and their cooccurrence within units of analysis suggests that YouTubers creating apomediated content use the same strategies as other popular influencers to gain visibility in this platform and to create an idea of a sustained relationship and intimacy with viewers through direct requests.

The prevalence of positive emotions across the sample suggests that YouTubers discussing Botox application in Spanish provide a message of joy (96%) and amazement (66%) related to this beauty procedure, and it may also relate to the general character of YouTube celebrity practices [4] and their relationship with viewers within Botox application videos and beyond, as part of their identity as YouTubers [27]. However, the expression of fear (64%) is also relevant, and it reflects a balance between the positive perspective of the content creator towards the beauty procedure and its results and a sense of fear towards possible risks it may entail. The emotional appeals, on the other hand, are overwhelmingly positive, with joy and amazement central and prevalent in the sample and signaling the emotions that viewers are expected to hold with respect to this content. These results reflect the use of specific emotive enunciation strategies that these YouTubers use to establish a relationship with their viewers, providing further insights to respond to RQ1 related to the strategies that are used most often for relationship-building between YouTubers and viewers.

### 6.2. Genre

The genre of the videos is closely related to the type of relationship YouTubers aim to establish with their viewers, providing a more nuanced answer to RQ1. The videos within the sample present a combination of genres, with complete tutorials (94%) that include a description of the necessary products (98%). Commercial content is present in the form of promotions about specific centers or professionals (82%) and brands (40%) with explicit messages to sell a product (66%). Thus, it could be argued that a sort of combined genre of “commercial tutorial” is prevalent in this sample, where “how to” messages converge with branding and marketing strategies to respond to questions of how, where, and who can perform this procedure.

Merely informational videos are scarce (16%), suggesting that YouTubers within this sample create content that is more involved and detailed, appealing to viewers to act (whether this action includes buying certain products or acquiring the services of certain centers and professionals for Botox-related services). The interactive nature of these videos resembles the interdiscursive character of YouTube beauty tutorials [29].

### 6.3. Credibility

In this section, we aim to connect our results to RQ2, related to the different approaches of these YouTubers to establish their credibility. Thus, within the credibility dimension, personal experience is present in 100% of the videos, suggesting that the relationship established by the YouTuber with viewers and followers is personal and calls for credibility from the personal experience of the creator, as YouTube users often search for health information featuring personal experiences [25,29,30]. Moreover, the inclusion of an external expert in 80% of the videos and 84% of them featuring mentions of expertise and training suggests that these YouTubers attempt to strengthen their own experiential credibility through expert advice or statements of expertise. This is significant in light of research that suggests that social media platforms such as YouTube negatively affect traditional expectations of expertise, providing beauty YouTube influencers with opportunities to show expertise by stating their knowledge of the products used [31].

In terms of RQ4b, related to the differences in terms of interactions among videos that display credibility strategies, a result of interest is the significant negative correlation between likes and mentions of relevant training and/or qualifications, which suggests that there may be a preference for personal experiences and user-generated content rather than expert advice and supports previous research that suggests that misleading health content is often the most viewed on this platform [18].

Successful previous experiences are present in 60% of the videos, establishing an additional dimension of credibility through the success and effectiveness of preceding procedures. The presence of reliable sources of information in 74% of the videos suggests that at least a portion of the content is useful, as described in previous research [6].

In terms of informational bias, more than half of the videos (64%) offer balanced information, featuring possible risks and benefits of this procedure, which supports previous studies about Botox YouTube videos [6]. The uncertain nature of much of the information provided (74%) suggests that these YouTubers are careful to offer decisive opinions despite the positivity expressed in other categories.

### 6.4. Attitude towards and Information about BTX

In response to RQ3, the generally positive attitude towards Botox within the sample is reflected by 92% of the videos that include positive aspects of this procedure and/or product, which resembles findings about the positive aspects present in cosmetic surgery videos on the platform [7], while only 42% portray a negative attitude towards it. Moreover, although 90% of videos describe how to apply the product, none of the videos in the sample are explicitly directed to professional audiences, which suggests that this platform is a prime context for potential Botox users to learn more about this procedure, the products available, and results, without an excessive focus on contraindications (26%), risks (34%), or care needed after application (20%). This may be due to the commercial nature of many of the videos and the interest in the promotion of services, brands, and products related to Botox application.

## 7. Conclusions

The undeniable role of social media in health information is reflected in the wide availability of apomediated content in the platform. In this sense, the present study provides insights into Botox tutorials on the platform with results that suggest that influencer culture converges with medical content and beauty procedures. Thus, Spanish-speaking YouTubers within our sample offer a combination of commercial tutorial content and interaction-seeking strategies that characterize contemporary influencer cultures. The positivity of their Botox-related content illustrates the nature of such procedures, reflecting a lack of content focusing on the care necessary beyond the procedure in most cases.

There is great interest in the international scientific community in studying apomediation and health-related content by non-health personnel and, consequently, many documents emerge in databases (WoS and Scopus) with the above-mentioned search criteria. The novelty of this research is based on three pillars: (1) It creates and validates in construct and content a model of qualitative analysis of health-related content (in this case, Botox, but it may be applicable to other scenarios). (2) It explains the narrative strategies used by non-health professional YouTubers, who seek to generate emotions in the audiences, with the use of enunciative emotions. (3) The different resources are used by these content creators to build credibility.

This has important implications with regard to media configurations of influence that permeate specialized medical realms. The widespread interest in beauty and especially procedures involving Botox means that this content will continue to be produced and refined to achieve commercial and popularity-related goals. In this sense, the present study provides some initial insights into the characteristics of this content that may be useful for practitioners of health literacy, medical professionals, and YouTube users.

The limitations of our study stem from its exploratory nature, which enables the identification of initial insights but requires further confirmation through additional testing and assessment. However, the present study provides a few areas for future inquiry.

Future lines of research can focus on the user’s perspective towards this type of content and how the different interactions and credibility strategies are perceived by users and viewers. The relationship between likes and expertise is also relevant, as well as a closer assessment of the credentials and actual expertise of those presenting the information. The ability of YouTube users to assess this content is also relevant for future research, as YouTubers are able to reach followers directly, bypassing traditional media gatekeepers and filters.

## Figures and Tables

**Table 1:** Elements of content evaluation on the analysis sheet.

Dimension	Category	Indicator	*k*
General information		Link to video; Name of YouTube Channel; Title of the video; Duration; Date	1.000
Interactions		Number of followers; Likes, Dislikes; Views; Comments (#)	1.000
Contact Enunciation	Target Audience	General population; Professionals	0.890
	Relationship with the camera	Absence on camera but the voice can be heard; On-camera presence and voice but not addressing the camera; Looks and speaks directly into the camera.	0.770
	Relationship with the public on an individual basis (individuation)	Use of the second person (you, they) and its variations; Direct request for comments and/or opinions; Use of the name of a follower; Response to a specific follower’s comment; Use of phrases to promote subscriptions; Invitation to watch other videos in the channel; Invitation to visit accounts on other platforms	0.682
Emotive enunciation	Emotional Expression (EE)	Joy; Sadness; Anger; Shame; Frustration; Amazement; Fear; Disgust; Envy	0.720
	Emotional Appeal (EA)	Joy; Sadness; Anger; Shame; Frustration; Amazement; Fear; Disgust; Envy	0.682
Genre	Tutorial	Explanation of a process from start to finish; Information about the products necessary to carry out the procedure	0.682
	Promotional Video	Inclusion of a brand; Mention of a specific center or professional; Inclusion of promotional messages to sell a product	0.682
	Informational	Mention of information without appealing to the user to take any action	0.770
	Interview	Inclusion of a person external to the channel in interview format	0.682
	Vlog	First-person content development	0.770
Strategies to establish content credibility and reliability	Expert guest	Inclusion of an expert on the subject	0.720
	Personal Expertise	Mention of relevant training or qualifications; Mention of having had experience with successful cases; Mention of publications on the subject	0.720
	Personal Experience	Explanation that the information is based on the personal experience of the YouTuber	0.770
	Objective information	Balanced Information; Biased information	0.890
	Additional sources	Inclusion of additional (reliable) sources; Inclusion of additional sources (unreliable or impossible to know)	0.890
	Areas of uncertainty	Mention of aspects about which the available information is uncertain	0.720
Attitude towards BTX	Positive	Description of positive aspects of the product	0.720
	Negative	Description of negative aspects of the product	0.682
	Mixed	Inclusion of positive and negative aspects of the product	0.682
Specific content about BTX	Action mechanism	Explanation of how the product works	0.720
	Procedure	Description of how to apply the product (for professionals); Description of how the product is applied (intended for self-application); Use of application “maps”	0.890
	Benefits and results	Description of what to expect	0.770
	Risks and contraindications	Description of side effects; Description of contraindications; Description of possible risks	0.770
	Self-care after product use	Instructions; Mention of things that can be done; Mention of things that should not be done	0.770

**Table 2:** General variables.

	*n*	Minimum	Maximum	Mean	SD	Skewness	Std. Error	Kurtosis	Std. Error
Number of followers	49	799	6,530,000	924,595.90		2.558	0.340	6.802	0.668
Duration (minutes)	50	6:16	33:17:00	15:03	6:05	1.304	0.337	1.711	0.662
Like	50	397	136,444	13,389.12	29,069,475	3.432	0.337	11.607	0.662
Dislikes	50	6	9184	642.68	1,697,490	4.400	0.337	19.555	0.662
Views	50	15,512	4,873,715	412,584.64	830,935,137	3.877	0.337	17.603	0.662
Comments	50	0	10,656	956.20	2,056,505	3.786	0.337	14.655	0.662
Valid N (listwise)	49								
